# Altered choroid plexus gene expression in major depressive disorder

**DOI:** 10.3389/fnhum.2014.00238

**Published:** 2014-04-22

**Authors:** Cortney A. Turner, Robert C. Thompson, William E. Bunney, Alan F. Schatzberg, Jack D. Barchas, Richard M. Myers, Huda Akil, Stanley J. Watson

**Affiliations:** ^1^Molecular and Behavioral Neuroscience Institute, University of MichiganAnn Arbor, MI, USA; ^2^Department of Psychiatry, University of MichiganAnn Arbor, MI, USA; ^3^Psychiatry and Human Behavior, University of California - IrvineIrvine, CA, USA; ^4^Department of Psychiatry and Behavioral Sciences, Stanford UniversityPalo Alto, CA, USA; ^5^Department of Psychiatry, Weill Cornell Medical College, Cornell UniversityIthaca, NY, USA; ^6^HudsonAlpha Institute for BiotechnologyHuntsville, AL, USA

**Keywords:** cytoskeleton, mRNA, hippocampus, depression, brain

## Abstract

Given the emergent interest in biomarkers for mood disorders, we assessed gene expression in the choroid plexus (CP), the region that produces cerebrospinal fluid (CSF), in individuals with major depressive disorder (MDD). Genes that are expressed in the CP can be secreted into the CSF and may be potential biomarker candidates. Given that we have previously shown that fibroblast growth factor family members are differentially expressed in post-mortem brain of subjects with MDD and the CP is a known source of growth factors in the brain, we posed the question whether growth factor dysregulation would be found in the CP of subjects with MDD. We performed laser capture microscopy of the CP at the level of the hippocampus in subjects with MDD and psychiatrically normal controls. We then extracted, amplified, labeled, and hybridized the cRNA to Illumina BeadChips to assess gene expression. In controls, the most highly abundant known transcript was transthyretin. Moreover, half of the 14 most highly expressed transcripts in controls encode ribosomal proteins. Using BeadStudio software, we identified 169 transcripts differentially expressed (*p* < 0.05) between control and MDD samples. Using pathway analysis we noted that the top network altered in subjects with MDD included multiple members of the transforming growth factor-beta (TGFβ) pathway. Quantitative real-time PCR (qRT-PCR) confirmed downregulation of several transcripts that interact with the extracellular matrix in subjects with MDD. These results suggest that there may be an altered cytoskeleton in the CP in MDD subjects that may lead to a disrupted blood-CSF-brain barrier.

## Introduction

The choroid plexus (CP) is composed primarily of capillary beds, the pia mater and a large number of epithelial cells. The CP produces cerebrospinal fluid (CSF), removes byproducts, plays a role in neuroendocrine signaling and provides structural support for the brain (Skipor and Thiery, 2008; Wolburg and Paulus, 2010). Yet to date, no human post-mortem gene expression studies of the CP have been published.

As the CP is found largely in the lateral ventricles, it is often co-dissected with surrounding brain tissue. This is particularly evident in dissections of the human hippocampus, a brain structure known to respond to antidepressants and play a role in neurogenesis (Cameron and McKay, 2001; Mallei et al., 2002; Duman, 2004; Bachis et al., 2008). Given the proximity of the CP in the lateral ventricle to the hippocampus and the ability of the CP to secrete proteins into the CSF that can act on the hippocampus, it is surprising that this structure has not been previously studied in individuals with mood disorders. The CP has, however, been shown to exhibit alterations following chronic stress in rats, a model known to induce depression-like behavior (Sathyanesan et al., 2012).

It is also important to consider the disease relevance of the source material. We do not know, for example, whether proteins found in the CSF are related to gene expression in the CP or are derived from other brain areas. Previous studies have reported differences in growth factor levels in the CSF (Kahl et al., 2009; Kiec-Wilk et al., 2010; Takebayashi et al., 2010). However, conflicting evidence exists that differential expression of a growth factor in gray matter did not translate to detectable differences in expression or levels in the blood or CSF (Lanz et al., 2012). This suggests that growth factors in the CSF might arise from many different tissue sources, including the CP.

Although nothing is known about gene expression in the CP in MDD subjects, a significant amount of literature has previously focused on the role of the hippocampus. For example, there is evidence for altered gene expression in the human post-mortem hippocampus in individuals with MDD (Sequeira et al., 2007, 2009). However, gene expression studies should be interpreted with caution, as hippocampal dissections also likely contain CP. In the present study, we used laser capture microscopy to selectively isolate the CP from control and MDD post-mortem human brain prior to assessment of gene expression using microarrays. An unbiased approach was also used to determine the top network and functions that may be altered in the CP in subjects with MDD. A subset of mRNAs differentially expressed by microarray analysis was then validated by qRT-PCR. This study was the first to determine gene expression in the CP in normal controls. We also observed differences in gene expression between normal controls and individuals with MDD.

## Methods and materials

### Post-mortem tissue quality and demographics

All human brains were obtained from the Brain Donor Program at the University of California—Irvine, and the studies were approved by both the University of California—Irvine and the University of Michigan Institutional Review Boards. Written informed consent was obtained from the next-of-kin of the deceased. Information regarding diagnosis, treatment and other clinically relevant variables were obtained from medical records, the coroner's investigation and family interviews. Table 1 shows a list of subject demographics for controls and individuals with MDD used in this study. Six subjects per group were used for microarray analyses, and an additional four subjects per group were added for the qRT-PCR validation study. No significant differences in age, gender, post-mortem interval or brain pH were detected between groups. All subjects also had an agonal factor score (Gustafsson et al., 2003) of 0. Previous studies found that AFS and brain pH are the strongest factors influencing gene expression (Li et al., 2004). Therefore, all brains used in this study had a pH greater than 6.6. The brains were removed at the time of autopsy, cooled to 4°C and then sliced into 0.75 cm coronal slabs which were then immediately stored at −80°C to preserve integrity (Jones et al., 1992). The slabs were then manually dissected while kept frozen at −80°C by trained staff into various brain region blocks, including the hippocampus which was used for this study.

**Table 1:** **Demographics for choroid plexus samples included in the mircorarray and qRT-PCR studies**.

**ID**	**Diagnosis**	**Age**	**Sex**	**Brain pH**	**Race**	**PMI**	**Cause of death**	**SSRI**
**1834**	**Control**	**40**	**M**	**6.76**	**C**	**12.3**	**LTMC**	**NO**
**2619**	**Control**	**48**	**M**	**6.79**	**C**	**20.2**	**SMC**	**NO**
**2805**	**Control**	**45**	**M**	**6.86**	**C**	**21.0**	**SMC**	**NO**
**3520**	**Control**	**74**	**F**	**7.21**	**C**	**18.5**	**SMC**	**YES**
**3572**	**Control**	**49**	**M**	**6.68**	**C**	**27.5**	**SMC**	**NO**
**4327**	**Control**	**56**	**M**	**6.64**	**C**	**9.0**	**SMC**	**NO**
2292	Control	55	M	6.89	C	15.0	SMC	NO
2248	Control	64	F	6.83	C	19.3	SMC	NO
3519	Control	65	M	6.88	AA	13.5	SMC	NO
4350	Control	68	M	6.67	AA	25.9	SMC	NO
**2267**	**MDD**	**19**	**M**	**7.11**	**C**	**18.0**	**SUICIDE**	**NO**
**2944**	**MDD**	**52**	**M**	**6.82**	**C**	**16.0**	**SMC**	**YES**
**3071**	**MDD**	**49**	**M**	**7.00**	**C**	**31.0**	**UNDETERMINED**	**YES**
**4260**	**MDD**	**48**	**F**	**6.62**	**C**	**12.0**	**UNDETERMINED**	**NO**
**4323**	**MDD**	**53**	**M**	**6.75**	**C**	**33.5**	**SMC**	**NO**
**4326**	**MDD**	**59**	**F**	**7.15**	**C**	**15.0**	**ACCIDENT**	**NO**
3169	MDD	35	M	7.04	C	24.75	ACCIDENT	YES
2315	MDD	58	M	6.93	C	24.0	SUICIDE	YES
2208	MDD	72	F	7.13	C	21.0	SUICIDE	YES
4272	MDD	72	F	6.61	C	27.7	SUICIDE	YES

Abbreviations: F, female; M, male; MDD, major depressive disorder; C, Caucasian; AA, African American; PMI, post-mortem interval; LTMC, long-term medical condition; SMC, sudden medical condition; SSRI, selective serotonin reuptake inhibitor (at the time of death).

Bold represents subjects used in the microarray study.

All subjects in the cohort were well-matched for age, gender, post-mortem interval, agonal factor and pH and used in the qRT-PCR study.

Averages ± s.e.m. are: age—control (56.4 ± 3.5), MDD (51.7 ± 5.0); pH—control (6.8 ± 0.5), MDD (6.92 ± 0.06); PMI—control (18.2 ± 1.86), MDD (22.3 ± 2.26); level—control (5.0 ± 0.02), MDD (4.9 ± 0.2).

### mRNA in situ hybridization

Previously dissected blocks from the post-mortem human hippocampus were sectioned (−20°C) at 10 μm and stored at −80°C. One section was placed onto a SuperFrostPlus slide (Thermo FisherScientific, Waltham, MA) and stored at −80°C until processing. Every 50th section was selected for mRNA *in situ* hybridization throughout the hippocampus, as previously described (Lopez-Figueroa et al., 2004). Briefly, slides were fixed in 4% paraformaldehyde, rinsed in 2X SSC, incubated in an acetic anhydride/triethanolamine solution and dehydrated. Sections were then hybridized with ^35^S-UTP and ^35^S-CTP cRNA probes overnight at 55°C. The next day, slides were washed, rinsed in increasing stringency of SSC, dehydrated and exposed to Kodak Biomax MR film (Eastman Kodak, Rochester, NY). Exposure time was empirically determined using test slides to visualize the region of interest. The cRNA probes were synthesized from human cDNA cloned in-house. The GAD67 (NM_000817, 738-964) cRNA probe was used to determine the anatomical level of the human hippocampus, and the transthyretin (TTR; NM_000371, 477-701) cRNA probe was used to visualize the CP. *In situ* hybridization autoradiograms were then digitized and scanned using the ScanMaker 1000XL Pro Flatbed Scanner (Microtek, Carson, CA) and SilverFast Ai Imaging software (LaserSoft Imaging, Sarasota, FL).

### Laser capture microscopy (LCM)

**Figure 1:**
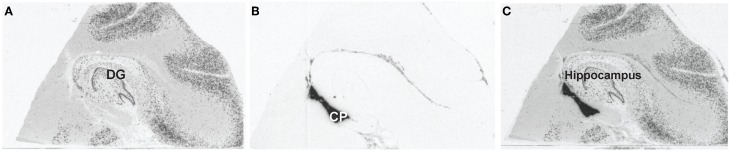
**Identifying the choroid plexus by mRNA *in situ* hybridization. (A)** Representative image of GAD67 gene expression to identify the hippocampal level for each subject. The dentate gyrus is the C-shaped structure in this image depicting level 5 of the hippocampus. **(B)** Representative image of TTR gene expression used to identify the choroid plexus for each subject. The choroid plexus is the dark region ventral to the hippocampus. **(C)** Representative image of GAD67 and TTR overlays.

**Figure 2:**
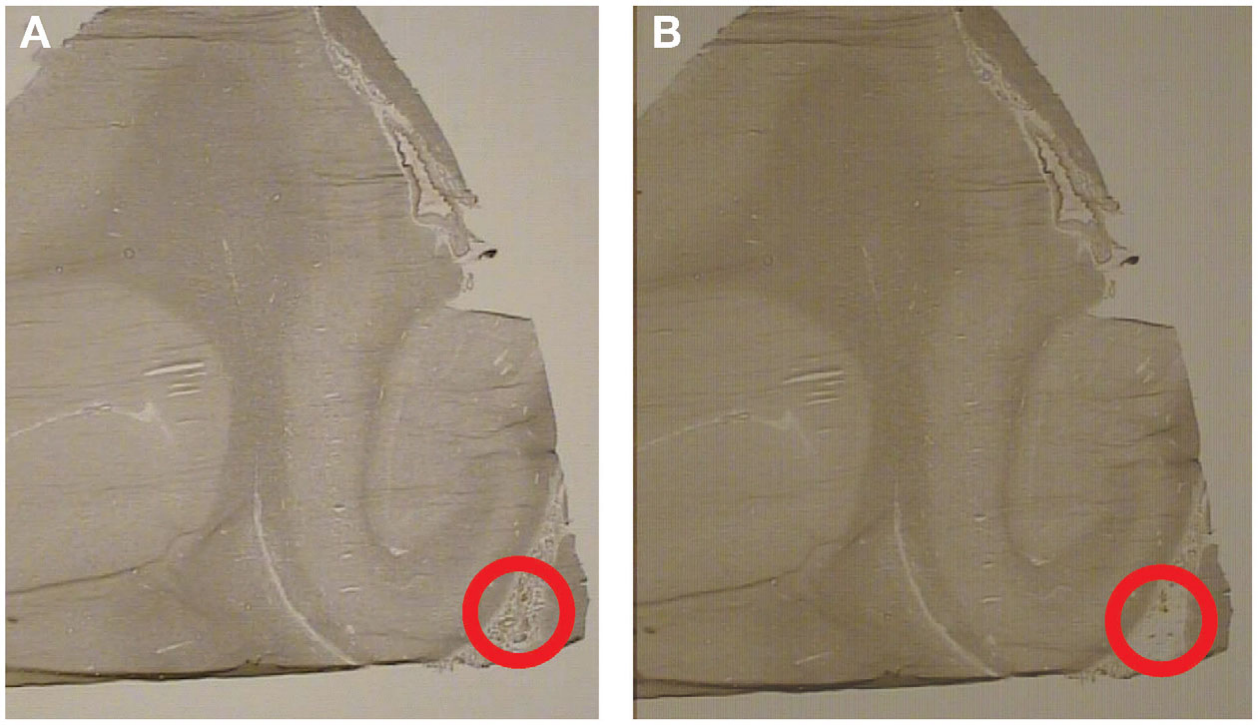
**Laser-capture microscopy images from the same subject before and after capture.** Representative images from the laser capture microscope (4X). The hippocampus is to the left and the choroid plexus within the lateral ventricle is located inside the circle. **(A)** Representative image of the choroid plexus before laser capture. **(B)** Representative image of the choroid plexus after laser capture. Notice that the choroid plexus has been removed from the area inside the circle.

The hippocampus was identified using GAD67 mRNA *in situ* hybridization, see Figure 1. The hippocampus was identified based on the C-like shape of the dentate gyrus. Transthyretin has been shown from mouse studies to be highly abundant in the CP and can be used as a reliable mRNA marker of this tissue (Marques et al., 2011). Sections containing the CP were processed through the following dehydration protocol: room temperature for 30 s, 75% ethanol for 30 s, dH_2_0 for 30 s, 75% alcohol for 30 s, two 95% alcohol washes for 30 s, 100% alcohol for 30 s, two xylene washes for 5 min each, and left to air dry for 20 min. Laser capture microscopy of the CP was performed at the level of the caudal hippocampus (levels 4–6, based on Amaral and Insuasti, 1990). Two field-of-views of the CP from one section was captured using the AutoPix LCM system (Arcturus, Mountain View, CA) onto CapSure Macro LCM caps (Arcturus, Mountain View, CA). Laser settings were set to 70 mW and 1.5 ms. Figure 2 shows a low magnification image (4X) of the CP before and after capture (area circled in red). The large blood vessels were not captured resulting in a sample of primarily epithelial cells.

### RNA isolation and amplification

Total RNA was extracted from adherent cells (on LCM caps) using the PicoPure RNA isolation kit (Arcturus, Mountain View, CA). Cell extracts were stored at −80°C between RNA extraction and final isolation. An optional DNAse treatment was used in the isolation process. RNA was eluted in a final volume of 11 μl. Quality and quantity of cRNA was determined on the Agilent 2100 Bioanalyzer using Picochip kits (Agilent, Palo Alto, CA). The quality of 18S and 28S peaks were determined as previously described (Bernard et al., 2011). For an average area of 2.7 mm^2^, we obtained an average concentration of 1 ng/μl and RNA quality was determined as previously described (Kerman et al., 2012). This process yielded an average of 11 ng of total RNA. The total isolated RNA was then amplified by the RiboAmp Plus 1.5-round RNA Amplification kit (Molecular Devices, Sunnyvale, CA). The final biotin-labeled cRNA was generated with the Bioarray High Yield RNA transcription kit (ENZO life sciences, Plymouth Meeting, PA). The quantity was determined on the Nanodrop ND-1000 spectrophotometer (Thermo Fisher Scientific Inc., Waltham, MA). After amplification, the total biotin-labeled cRNA was an average of 5 μg per sample.

### Microarray analysis

Equal amounts (750 ng) of amplified total RNA sample from each human sample was hybridized to HumanHT-12v4.0 Expression BeadChips and scanned on the BeadStation system (Illumina Inc., San Diego, CA) following the manufacturer's instructions (Turner et al., 2011). Each Beadchip provides coverage of more than 47,000 probes. The microarray data was quantile normalized in BeadStudio using Illumina's error model (Illumina Inc., San Diego, CA). Fold-changes and *p*-values were calculated from the average array signal. Data were also analyzed by Ingenuity Pathways Analysis Software v8.0 (Ingenuity©Systems, Redwood City, CA). The Functional Analysis tool identified biological functions and/or diseases that were most significant to the data set. Molecules from the dataset that had a fold-change cutoff of 1.1, a *p*-value less than 0.05 and were associated with biological functions and/or diseases in Ingenuity's® Knowledge Base were considered for the analysis. To follow-up on the top pathway identified by Ingenuity Pathway Analysis, we evaluated gene ontology using Cytoscape v2.8.2 with a BinGO plug-in.

### qRT-PCR

Table 2 shows a list of primers used for qRT-PCR validation. Amplified cRNA (1 μ g) was reverse transcribed using the iScript cDNA synthesis kit (Bio-Rad Laboratories, Hercules, CA) in a total reaction volume of 20 μ l. cDNA (1 μl) was used as the template for real-time PCR assays with a MyiQ real-time PCR system (Bio-Rad Laboratories). The quantitative PCR was conducted in duplicate using iQ SYBR Green Supermix, according to the manufacturer's instructions (Bio-Rad Laboratories). Relative expression of the gene of interest was normalized to β-actin expression in each sample. It is important to note that β-actin did not differ between controls and individuals with MDD. The expression level of the gene of interest was evaluated using the 2^−(ΔΔ*Ct*)^ method and values for each gene were expressed as fold-changes (Livak and Schmittgen, 2001). The PCR product quality was monitored using post-PCR melt-curve analysis at the end of the amplification cycles.

**Table 2:** **List of primers used in the qRT-PCR study**.

**Accession no.**	**Symbol**	**Primers (forward, reverse)**	**Length (bp)**
NM_001004019.1	FBLN2	GACTCCTGTGGCTTCTGGAC	164
		CGTGTCTCTGGTCCTCAGGT	
NM_002477.1	MYL5	CTGTTTGGGGAGAAGCTGAG	120
		CATCAGCAGACGCTTGATGT	
NM_002474.1	MYH11	GGGGAGAAAGTCACCGAAAT	143
		AACTGTGCGTGTCTGAGGTG	
NM_002473.3	MYH9	GCCACCTGCACAGGTATTTT	196
		TGCCGTAAGTCTCAATGCAG	
NM_002470.1	MYH3	GAGGAGGCTGATGAACAAGC	148
		TCCTGCTGGAGGTGAAGTCT	
NM_005767.3	P2RY5	AAATTGGACGTGCCTTTACG	116
		TAACCCAAGCACAAACACCA	
NM_004137.2	KCNMB1	GTGAAGTCATTGCCTGCTCA	180
		GGAGAACTCAGGCACAGAGG	
NM_138957.2	MAPK1	CCAGACCATGATCACACAGG	163
		CTGGAAAGATGGGCCTGTTA	

Abbreviations: FBLN2, fibulin 2, transcript variant 1; MYL5, myosin, light chain 5, regulatory; MYH11, myosin, heavy chain 11; MYH9, myosin, heavy chain 9; MYH3, myosin, heavy chain 3; P2RY5, purinergic receptor P2Y, G-protein coupled, 5; KCNMB1, potassium large conductance calcium-activated channel, subfamily M, beta member 1; MAPK1, mitogen-activated protein kinase 1, transcript variant 1.

### Statistical analyses

For qRT-PCR, a Student's *t*-test was performed using SPSS (IBM, Armonk, NY). For pathway analysis, a right-tailed Fisher's exact test was used to calculate a *p*-value determining the probability that each biological function and/or disease assigned to that data set is due to chance alone. For gene ontology, Benjamini & Hochberg false discovery rate (FDR) correction was applied to the dataset.

## Results

### Controls

The most highly expressed known transcript in the human post-mortem CP was transthyretin (TTR). The primary role of TTR is to transport thyroxine (T4) and retinol in the brain (Fleming et al., 2009). However, TTR also has proteolytic activity (e.g., neuropeptide Y and β-amyloid). TTR protein levels had previously been associated with depression, such that low levels of TTR have been correlated with high suicidal ideation and low 5-HIAA (Sullivan et al., 2006). A previous study also found TTR to be downregulated in the CSF of individuals with MDD (Ditzen et al., 2011). However, TTR gene expression was not altered in the CP in MDD subjects in our study.

Supplementary Table 1 lists the 11,506 transcripts that were significantly detected across all samples in the control CP. Notably, half of the 14 mostly highly expressed transcripts were transcripts that encode ribosomal proteins. However, none of these transcripts were altered in subjects with MDD.

### Subjects with MDD

In general, there were 169 transcripts differentially expressed between MDD subjects and controls, see Supplementary Table 2 for the complete list. The majority of these transcripts (75%) were downregulated with only a small percentage of transcripts upregulated (25%). The fact that the majority of the transcripts were downregulated is similar to what we have observed in the hippocampus in subjects with MDD (unpublished observations).

**Figure 3:**
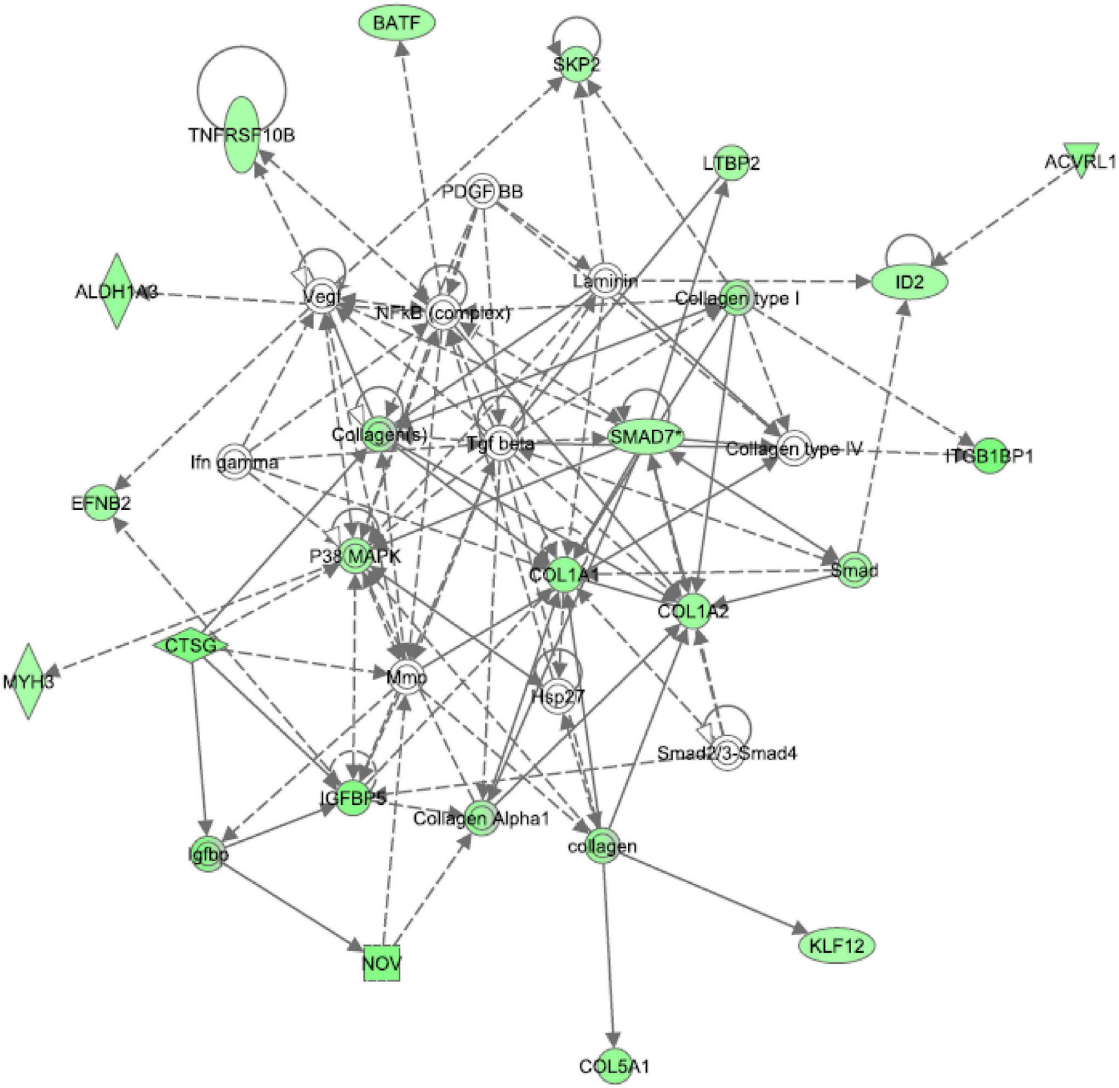
**Top network identified from Ingenuity pathway analysis in the choroid plexus of MDD subjects.** TGFβ is at the center of the top network dysregulated in MDDs. All of the transcripts in this network were downregulated in MDDs.

When Ingenuity pathway analysis was performed on the dataset, the top five significant functions were connective tissue disorder, dermatological diseases, developmental disorder, genetic disorder, and metabolic disease. The top two significant pathways were hepatic fibrosis and calcium signaling. The top network focused on transforming growth factor-beta (TGFβ). As shown in Figure 3, all of the transcripts associated with the TGFβ network were downregulated in MDD subjects compared to controls. Gene ontology analysis confirmed that growth factor binding (corrected *p*-value: 0.00885) was the most significant function in this dataset. Indeed, seven members of the TGFβ network validated by this analysis. TGFβ signaling is known to play a role in cytoskeletal dynamics and actin reorganization (Baghdassarian et al., 1993; Gagelin et al., 1995). Since TGFβ is associated with various components of the extracellular matrix, we decided to interrogate transcripts with high fold-changes related to structural support and integrity, some of which are part of the TGFβ pathway.

We selected eight transcripts for real-time PCR validation, see Table 3. Since we can predict the direction of change based on the microarray results, we performed one-tail *t*-tests for qRT-PCR. Three transcripts were confirmed by qRT-PCR and two transcripts exhibited non-significant trends in the same direction as the microarray results. We will describe these five transcripts in the next few paragraphs. Fibulin 2 (FBLN2) was significantly decreased in subjects with MDD compared to controls. FBLN2 is a secreted glycoprotein that is produced by epithelial cells and can bind calcium. In terms of function, FBLN2 interacts with many other proteins, including the laminins and integrins, to stabilize the extracellular matrix (Zhang et al., 1996; de Vega et al., 2009). It is possible that downregulation of this transcript may lead to destabilization of the extracellular matrix.

**Table 3:** **Results of the microarray and qRT-PCR experiments in MDDs**.

**Accession no.**	**Symbol**	**Microarray**		**qRT-PCR**	
		**Fold-change**	***P*-value**	**Fold-change**	***P*-value**
NM_001004019.1	FBLN2	−2.75	**0.014**	−3.18	**0.026**
NM_002477.1	MYL5	1.44	**0.014**	1.43	0.115
NM_002474.1	MYH11	−3.46	**0.038**	−3.08	**0.025**
NM_002473.3	MYH9	−1.43	**0.021**	1.71	0.131
NM_002470.1	MYH3	−1.33	**0.015**	−1.16	0.271
NM_005767.3	P2RY5	−1.48	**0.049**	*−1.41*	*0.057*
NM_004137.2	KCNMB1	−1.71	**0.031**	−3.01	**0.027**
NM_138957.2	MAPK1	−1.35	**0.027**	*−2.25*	*0.088*

Abbreviations: FBLN2, fibulin 2, transcript variant 1; MYL5, myosin, light chain 5, regulatory; MYH11, myosin, heavy chain 11; MYH9, myosin, heavy chain 9; MYH3, myosin, heavy chain 3; P2RY5, purinergic receptor P2Y, G-protein coupled, 5; KCNMB1, potassium large conductance calcium-activated channel, subfamily M, beta member 1; MAPK1, mitogen-activated protein kinase 1, transcript variant 1.

Fold-changes indicated fold change in the MDD group compared to the control group.

Significant (p < 0.05) changes are indicated in bold; those that exhibited a trend toward significance (p < 0.1) are italicized.

A myosin heavy chain transcript and a calcium-activated potassium channel were also altered in individuals with MDD. Myosin heavy chain 11 (MYH11) was significantly decreased in MDD subjects compared to controls. This myosin motor can bind actin and play a role in ATP hydrolysis (Renard et al., 2011; Armstrong et al., 2012). Thus, the actin cytoskeleton might be altered in subjects with MDD. The calcium-activated and voltage-dependent potassium channel β1 subunit (KCNMβ1) was also significantly decreased in individuals with MDD. Although not much is known about the role of this large conductance channel in the brain, it has been linked to cardiovascular disease and hypertension in the periphery (Grimm et al., 2009). However, it is plausible that this channel also may play a role in filtration, as total gene expression in the kidney is similar to that of the CP suggesting that the two tissues may share similar functions (Sathyanesan et al., 2012). This particular subunit of the channel is also the regulatory subunit of the channel and may alter surface expression of the receptor (Toro et al., 2006).

There were also non-significant trends for mitogen-activated protein kinase 1 (MAPK1) and the purinergic receptor, P2YR5, to be altered in MDD subjects. MAPK is part of the TGFβ network (see Figure 2) and is known to play a role in tyrosine kinase receptor signaling. MAPK is also known to bind to and phosphorylate cytoskeletal proteins (Veeranna et al., 2000). Moreover, P2YR5 (aka LPAR6) plays a role in endothelial cell morphology and may be involved in actin reorganization, as it is a receptor for lysophosphatidic acid (Ishii et al., 2009). It is also possible that this receptor may regulate vascular permeability in the CP. In summary, all five of the transcripts from the qRT-PCR experiments were downregulated in subjects with MDD. Taken together, these results suggest that there may be an altered extracellular matrix or cytoskeleton in MDD subjects.

## Discussion

This study is the first to assess gene expression in the CP in MDD subjects, as well as in psychiatrically normal controls. Moreover, two salient pieces of information have emerged from this study. First, transcripts that encode ribosomal proteins were highly expressed in the CP in controls. Secondly, multiple transcripts that interact with the extracellular matrix and cytoskeleton were downregulated in individuals with MDD. Thus, there may be a disrupted CP in subjects with MDD.

In comparing our control data in humans to a previously published mouse study that used light microscopy dissection of the CP and microarrays, there was reasonable agreement among highly expressed transcripts (Marques et al., 2011). For example, there was a 25% overlap between our top 12 transcripts and their top 12 transcripts (excluding predicted sequences), including several members involved in energy demand (e.g., cox4i1 and atp5b). Moreover, this finding is in agreement with the high number of mitochondria known to exist in the CP (Cornford et al., 1997; Johanson et al., 2011). Interestingly, the P-glycoprotein pump (ABCB1) was not significantly detected in control tissue. This is likely because ABCB1 is located in the vascular endothelium near the epithelial cells of the CP (Mercier et al., 2004; Gazzin et al., 2008; Roberts et al., 2008; Kratzer et al., 2013).

This study identified transcripts that were differentially expressed between controls and MDD subjects in the CP. We did not find alterations in growth factors in the CP of MDD brains. However, several transcripts involved in calcium signaling were altered in the CP. Calcium signaling may represent another interesting area of therapeutic investigation in depression. We identified the TGFβ network to be identified by Ingenuity Pathway Analysis, and the significance of this pathway was confirmed by gene ontology analysis. The finding that the TGFβ network was altered suggests that less anti-inflammatory molecules may also be present in the CP of individuals with MDD. Further studies should be designed to follow-up on TGFβ signaling in subjects with MDD. Interestingly, MAPK1, a member of the TGFβ pathway, was the only one of the five interesting transcripts by qRT-PCR previously known to be altered in MDD brains. Single nucleotide polymorphisms in MAPK1 have been associated with treatment resistance and remission in depression (Calati et al., 2013). Furthermore, MAPK1 and related kinases have either reduced expression or phosphorylation in post-mortem brains of depressed individuals that committed suicide (Dwivedi et al., 2001, 2009).

It should be mentioned that there were limitations to this study. First, we only assessed the CP adjacent to the hippocampus. It is possible that the CP located in other regions of the brain may exhibit a different pattern of gene expression in MDD subjects. Moreover, we only assessed CP gene expression near the caudal hippocampus. This was due to the availability of high-quality sectioned material that also contained the CP. Interestingly, 40% of the depressed subjects included in this study committed suicide. It is possible that our results may not generalize to studies that use subjects with mild or moderate depression. Another limitation of the study was that we may have not been properly powered to validate some of the gene expression findings by qRT-PCR. To this end, we recognize that the findings from this study may not be particularly strong. Although we were limited by the number of high quality subjects available for this study, more subjects may be required to validate other transcripts changes, particularly those with lower fold-changes. It is important to note that we added new patients for the qRT-PCR study. Finally, the qRT-PCR primers did not always target to the same region as the microarray probe (e.g., MYH9). Thus, the specifics of the methodology helps explain why fewer transcripts than expected validated in this study.

The possibility that the extracellular matrix of the CP may be altered in depression has profound implications for identifying biomarkers in the CSF. Although many of the transcripts that were interrogated by qRT-PCR are not known to exist as proteins in the CSF, many of the collagens shown in Figure 3 are present as precursors or preproproteins (Bora et al., 2012). Since fibulin-2 (FBLN2) is a secreted protein that binds calcium and stabilizes the extracellular matrix, it would be interesting to determine whether FBLN2 may have mood altering effects and act as an antidepressant. Finally, since transcripts that interact with actin (i.e., MYH11, P2RY5) were also downregulated, there may be an altered cytoskeleton in MDD subjects.

In conclusion, the majority of the abundantly expressed transcripts in the CP are transcripts that code ribosomal proteins under normal physiological conditions. In subjects with MDD, several transcripts that interact with the extracellular matrix and cytoskeleton were decreased in the CP. Given the variety of functions performed by the CP and the identification of various transcripts linked to depression for the first time, the CP is a novel target for the development of therapeutics. With the majority of the transcripts downregulated, the structure of the CP is likely disrupted. Ways to boost the extracellular matrix or cytoskeleton should be beneficial in treating depression.

## Author contributions

Cortney A. Turner performed the study concept and design, laboratory procedures, data analysis, writing of the first draft of the manuscript and final revision of the manuscript. Robert C. Thompson performed data analysis, laboratory procedures and final revision of the manuscript. William E. Bunney supervised sample collection and final revision of the manuscript. Alan F. Schatzberg helped revise an earlier draft of the manuscript. Richard M. Myers assisted with an earlier version of the manuscript. Jack D. Barchas assisted with the final draft of the manuscript. Huda Akil supervised study design and manuscript revision. Stanley J. Watson supervised dissection of the material, study design and manuscript revision.

## Conflict of interest statement

The authors declare that the research was conducted in the absence of any commercial or financial relationships that could be construed as a potential conflict of interest.
